# Short-course Benznidazole treatment to reduce *Trypanosoma cruzi* parasitic load in women of reproductive age (BETTY): a non-inferiority randomized controlled trial study protocol

**DOI:** 10.1186/s12978-020-00972-1

**Published:** 2020-08-24

**Authors:** María L. Cafferata, María A. Toscani, Fernando Althabe, Jose M. Belizán, Eduardo Bergel, Mabel Berrueta, Edmund V. Capparelli, Álvaro Ciganda, Emmaria Danesi, Eric Dumonteil, Luz Gibbons, Pablo E. Gulayin, Claudia Herrera, Jeremiah D. Momper, Steven Rossi, Jeffrey G. Shaffer, Alejandro G. Schijman, Sergio Sosa-Estani, Candela B. Stella, Karen Klein, Pierre Buekens

**Affiliations:** 1 Instituto de Efectividad Clinica y Sanitaria (IECS), Buenos Aires, Argentina; 2 Unidad de Investigación Clínica y Epidemiológica Montevideo (UNICEM), Montevideo, Uruguay; 3 Maternal and Perinatal Health. UNDP-UNFPA-UNICEF-WHO-World Bank Special Programme of Research, Development and Research Training in Human Reproduction (HRP), Department of Sexual and Reproductive Health and Research (SRH), World Health Organization, Geneva, Switzerland; 4 Skaggs School of Pharmacy and Pharmaceutical Sciences, University of California, San Diego (UCSD), San Diego, USA; 5 Schools of Medicine, University of California, San Diego (UCSD), San Diego, USA; 6 Centro Nacional de Diagnóstico e Investigación en Endemoepidemias (CeNDIE) ANLIS Dr. C. G. Malbrán, Buenos Aires, Argentina; 7 School of Public Health and Tropical Medicine, Tulane University, New Orleans, USA; 8 Laboratorio de Biología Molecular de la Enfermedad de Chagas (LaBMECh), Instituto de Investigaciones en Ingeniería Genética y Biología Molecular “Dr. Héctor Torres” (INGEBI), Buenos Aires, Argentina; 9 Drugs for Neglected Diseases initiative – Latin America (DNDi), Rio de Janeiro, Brazil; 10 Centro de Investigaciones en Epidemiología y Salud Pública (CIESP-IECS), Consejo Nacional de Investigaciones Científicas y Técnicas (CONICET), Buenos Aires, Argentina

**Keywords:** Benznidazole, Chagas disease, Preconception care, Randomized controlled trial, *Trypanosoma cruzi*, Benznidazol, Enfermedad de Chagas, Cuidado preconcepcional, Ensayo clínico controlado aleatorizado, *Trypanosoma cruzi*

## Abstract

**Background:**

Retrospective observational studies suggest that transmission of *Trypanosoma cruzi* does not occur in treated women when pregnant later in life. The level of parasitemia is a known risk factor for congenital transmission. Benznidazole (BZN) is the drug of choice for preconceptional treatment to reduce parasitic load.

The fear of treatment-related side effects limits the implementation of the Argentine guideline recommending BZN 60d/300 mg (or equivalent) treatment of *T. cruzi* seropositive women during the postpartum period to prevent transmission in a future pregnancy. A short and low dose BZN treatment might reduce major side effects and increase compliance, but its efficacy to reduce *T. cruzi* parasitic load compared to the standard 60d/300 mg course is not yet established. Clinical trials testing alternative BZN courses among women of reproductive age are urgently needed.

**Methods and design:**

We are proposing to perform a double-blinded, non-inferiority randomized controlled trial comparing a short low dose 30-day treatment with BZN 150 mg/day (30d/150 mg) vs. BZN 60d/300 mg. We will recruit not previously treated *T. cruzi* seropositive women with a live birth during the postpartum period in Argentina, randomize them at 6 months postpartum, and follow them up with the following specific aims:

Specific aim 1: to measure the effect of BZN 30d/150 mg compared to 60d/300 mg preconceptional treatment on parasitic load measured by the frequency of positive Polymerase Chain Reaction (PCR) (primary outcome) and by real-time quantitative PCR (qPCR), immediately and 10 months after treatment.

Specific aim 2: to measure the frequency of serious adverse events and/or any adverse event leading to treatment interruption.

**Trial registration:**

ClinicalTrials.gov. Identifier: NCT03672487. Registered 14 September 2018

## Resumen (Spanish)

**Antecedentes:**

Estudios observacionales retrospectivos sugieren que la transmisión del *Trypanosoma cruzi* no ocurre en mujeres tratadas cuando quedan embarazadas más adelante. La carga parasitaria en la sangre es un factor de riesgo conocido para la transmisión congénita. El Benznidazol (BZN) es el fármaco de elección para el tratamiento preconcepcional de la mujer en edad fértil para reducir la carga parasitaria.

El temor a los efectos adversos relacionados con el tratamiento limita la implementación de la Guía Argentina que recomienda el tratamiento con BZN 60d/300 mg (o equivalente) en mujeres seropositivas a *T. cruzi* durante el período posparto para prevenir la transmisión en un futuro embarazo. Un tratamiento más corto y con una dosis menor de BZN podría reducir los eventos adversos y mejorar la adherencia al mismo, pero aún no se ha establecido su eficacia para reducir la carga parasitaria de *T. cruzi* en comparación con el esquema estándar de 60d/300 mg. Se necesitan con urgencia ensayos clínicos que prueben esquemas alternativos de BZN en mujeres en edad reproductiva.

**Métodos y diseño:**

Estamos proponiendo realizar un ensayo clínico controlado aleatorizado doble ciego de no inferioridad que compara un tratamiento corto de 30 días a dosis bajas con BZN 150 mg/día (30d/150 mg) versus BZN 60d/300 mg. Se reclutarán mujeres seropositivas para infección por *T. cruzi* no tratadas previamente, que hayan tenido un nacido vivo, durante el periodo de posparto en Argentina. Las mujeres serán aleatorizadas a los seis meses posparto y serán seguidas, con los siguientes objetivos específicos:

Objetivo específico 1: evaluar el efecto del tratamiento preconcepcional con BZN 30d/150 mg comparado con BZN 60d/300 mg por medio de la carga parasitaria, medida por la frecuencia de PCR positiva (resultado primario) y PCR cuantitativa a tiempo real (qPCR), al final del tratamiento y a los 10 meses post tratamiento.

Objetivo específico 2: medir la frecuencia de eventos adversos serios y/o cualquier otro evento adverso que conduzcan a la interrupción del tratamiento con BZN 30d / 150 mg en comparación con BZN 60d/300 mg.

## Plain English Summary

Chagas disease is caused by the parasite *Trypanosoma cruzi.* The chronic disease can last for decades and is a major cause of heart disease in the Americas. Around 6 million persons are currently infected, including 1 million women of reproductive age. Despite it is transmitted by a blood-sucking bug, a growing number of newborns get the infection from their mothers during pregnancy or birth (congenital transmission), and infected babies are at risk of developing chronic Chagas disease later in life.

Previous studies suggest that women treated at a young age achieve a long-lasting reduction of blood levels of the parasite and therefore do not transmit *Trypanosoma cruzi* when pregnant later in life. But doctors and patients are reluctant to prescribe or take the treatment of choice to prevent transmission in a future pregnancy, benznidazole 300 mg for 60 days, because they are afraid of side effects.

In this study, we propose to compare the standard treatment with an alternative one which reduces by 50% the dose and duration of treatment: 150 mg of benznidazole for 30 days. We will recruit women carrying the parasite in Argentina, not previously treated, with a live newborn. Six months after the delivery, they will be assigned by chance to either receive low-dose treatment or the standard one. Then, they will be followed up for a year to evaluate side effects, compliance to treatment and the amount of the parasite in the blood.

We have the expectation that the new alternative course will not be less effective than the conventional one, while diminishing side effects and costs.

## Introduction

### Statement of the problem

Chagas disease, or American trypanosomiasis, is caused by the protozoan parasite *Trypanosoma cruzi*. It is a major cause of cardiac disease in the Americas. An estimated 6 million persons are currently infected, including 1 million women of reproductive age [1]. In the U.S., the estimated number of *T. cruzi*-infected women of reproductive age was 130,522 in 2000 [2]. Mothers can transmit *T. cruzi* to their babies during pregnancy, and infected babies are at risk of developing chronic Chagas disease later in life [3]. Our meta-analysis of published data showed a 5.0% congenital transmission rate in endemic countries [4]. There is a risk of pregnancy complications, including preterm premature rupture of membranes and preterm delivery. *T. cruzi*-infected newborns may have severe morbidity and are at risk of Neonatal Intensive Care Unit (NICU) hospitalization and neonatal mortality [5]. Available drugs are not approved for use during pregnancy. Asymptomatic infected newborns can be effectively treated if detected early, but a follow-up to at least 8 months of age is needed in most cases to diagnose congenital transmission by measuring persisting antibodies [6]. Losses to follow-up are frequent, and many infected infants remain untreated. There is thus an urgent need to prevent congenital transmission of *T. cruzi*.

## Background

### Studies and current recommendations on treatment to prevent congenital transmission

Retrospective observational studies suggest that transmission of the *Trypanosoma cruzi* does not occur in treated women when pregnant later in life [7–9]. The level of parasitemia is a known risk factor for congenital transmission [5, 10–12]. Two retrospective observational studies suggested that women treated at a young age do not transmit *T. cruzi* when pregnant later in life [7, 9].

The first study included 32 children born to 16 women who were treated with BZN when they were six to 15 years old and who were evaluated 14 years later. None of the children were infected [9]. A more recent and larger observational study compared women treated before pregnancy to untreated women. On average, women were treated 17 years before follow-up. Among the 222 children born to untreated women, 34 were infected with *T. cruzi* (15.3%), while no infection was found among the 132 children of previously treated women [7]. Another small observational study found no congenital transmission among 15 women who became pregnant from one to 8 years after treatment [8].

We found no published or ongoing randomized controlled trials evaluating preconceptional etiological treatment of women infected with *T. cruzi* to prevent congenital transmission.

Despite the limited observational evidence and the lack of experimental studies, experts recommend or strongly suggest that *T. cruzi* seropositive women of reproductive age should be treated to prevent congenital transmission in future pregnancies [9, 13]. In Argentina, the national guideline recommends the treatment of *T. cruzi* seropositive women during the postpartum period with either BZN or Nifurtimox, with a preference for BZN due to the high frequency of side effects of Nifurtimox [14].

### Drug of choice for preconceptional treatment

BZN is the recommended trypanocidal drug for the prevention of congenital transmission. A Cochrane systematic review summarized the studies that evaluated the effects of trypanocidal drugs on parasite-related and patient outcomes [15]. BZN is the drug that was studied the most. Furthermore, the observational studies showing a potential preventive effect on congenital transmission evaluated BZN.

### Randomized controlled trials on BZN

The Cochrane review summarized four randomized controlled trials (RCTs), none of which were performed among women of reproductive age with PCR as an outcome [15]. Treatment with BZN showed between a 50 and 88% statistically significant reduction in parasite-related outcomes (e.g., positive serology, PCR).

Two additional trials were recently published. The CHAGASAZOL trial compared 60 days of BZN to posaconazole, and the intention-to-treat analysis showed 38.4% treatment failure measured by PCR in the BZN group vs. more than 80% treatment failure in the posaconazole groups [16]. The mean age of the CHAGASAZOL trial subjects in the BZN group was 40. The BENEFIT trial compared BZN vs. placebo among patients with Chagas’ cardiomyopathy [17]. The mean age of the subjects was 55 years. Positive conventional PCR was 60.5% at baseline, and the rate of conversion to negative PCR was 66.2% in the BZN group vs. 33.5% in the placebo group. In the Argentina and Bolivia sites, conversion rates to a negative PCR were 73.0% in the BZN group and 28.6% in the placebo group. There was no effect on the complications of cardiomyopathy. The BENEFIT trial is the largest placebo-controlled randomized trial performed thus far.

The comparison of treatment effects observed in trials in adults compared to those in children [15, 17] and observational evidence [7, 18] suggest that the therapeutic response to BZN treatment may be better and stronger in young and healthy subjects than in older subjects with organ compromise, like those who participated in the BENEFIT and CHAGASAZOL studies.

### BZN dosage and side effects

BZN usual dosage has been 5 mg per kg of body weight per day for 30 to 60 days [14]. This dosage is not free of side effects, including dermatitis, which usually occurs during the first weeks, and ocassionally peripheral neuropathy, which seems to be related to the cumulative dose and may take months to resolve [14, 19, 20]. Gastrointestinal effects, including vomiting and pain, are also frequent side effects that can be theoretically prevented by diet. Other severe adverse events, although infrequent, are bone marrow depression, toxic hepatitis, and lymphomas [20]. In the BENEFIT trial, the initial regimen of 5 mg per kg of body weight per day for 60 days was modified to a fixed dose of 300 mg per day for 40 to 80 days [17]. Of concern, the rate of treatment interruption because of an adverse event was 23.9% in the BZN group compared to 9.5% in the placebo group. Additionally, 13.4% of patients in the BZN group permanently discontinued treatment compared to 3.6% in the control group. Dermatitis, digestive intolerance, and neuropathy accounted for more than 90% of the interruptions [17]. Other studies suggest that the adverse events rate tends to be lower in children and young adults [13, 18, 20].

Minimizing side effects of BZN treatment is a priority, as it may prevent harm and increase compliance [14]. This is especially important for prevention of congenital transmission purposes in a population of infected but mostly still healthy young women. In Argentina, the fear of side effects limits the implementation of the guideline recommending BZN 60d treatment of *T. cruzi* seropositive women during the postpartum period to prevent transmission in a future pregnancy [14].

Do shorter BZN treatments reduce major side effects and increase compliance, compared to the standard 60d course? Thirty-day treatments have been used in non-randomized intervention studies, suggesting they might be as effective as a longer treatment and may prevent severe effects like peripheral neuropathy and bone marrow depression [21]. However, 30d courses may not prevent the most frequent adverse events like dermatitis and digestive intolerance.

Do lower dose BZN courses reduce major side effects, compared to the standard 5 mg/kg/d or 300 mg/d? Dermatologic effects seem to be idiosyncratic and not dose-related [20]. Pinazo and colleagues found no correlation between BZN levels and the incidence of adverse events in a population of 54 adult patients who were *T. cruzi* seropositive [22]. However, a pilot study evaluating a 5 mg/kg intermittent treatment for 60d showed that while adverse events were reported by 50% of the subjects, the severity of the effects was mostly mild and that treatment interruption occurred in only one subject. The authors concluded that reducing the dose by intermittent courses might reduce adverse events and that experimental studies are warranted [23]. Further evidence comes from PK studies in children and adults. Children show a lower incidence of adverse events than adults, but the mechanisms responsible for the age difference are still unclear; the enzymatic activity of nitro-reduction and generation of free radicals, which is low in children, as well as the higher clearance of BZN in children, with lower accumulation compared to adults, are among the postulated mechanisms [18, 20]. Altcheh and colleagues conducted a PK study in children and a model-based analysis, including adult data from previous BZN studies [18]. They observed that BZN concentrations in children were markedly lower than those reported in adults (treated with comparable mg/kg doses). Treatment was well tolerated with few adverse effects, a marked difference from adults. The authors suggest that it is conceivable that a BZN dose reduction for adults, which would result in systemic exposures similar to those in children, would not have a detrimental impact on treatment response and could lead to a reduction in the incidence of adverse events.

### BZN dosage and trypanocidal effect

No randomized controlled trial has been published thus far comparing the trypanocidal effect of courses shorter than 60 days, or courses with low BZN doses. A BZN 30d course with 5 mg/kg daily is generally accepted as a complete treatment in Argentina but is only supported by non-experimental evidence of non-inferiority compared to 60d [20].

There is indirect evidence supporting the potential efficacy of shorter courses from a cohort study in Argentina, following up 81 patients that permanently discontinued BZN treatment (5 mg/kg/d for 30 days) due to side effects. The median duration of treatment was 10 days, with 75% of the patients discontinuing before or at 15 days [24]. At follow-up at 9 years, 20% of these patients seroconverted in three *T. cruzi* serological tests and 39% of the patients seroconverted in only one test.

One interesting study in mice has shown that shorter courses, with as few as 13 doses of BZN at five-day intervals, perform equally well in cure rates than 40 daily doses. These results question the presumption that the effectiveness of BZN depends on sustaining a concentration above the minimum inhibitory concentration, suggesting that the effect may be produced through a “maximum concentration mechanism” with extended post-treatment effects and an impact on pathogen growth after complete elimination of the drug [25]. The authors also suggest that standard BZN protocols may be significantly overdosing patients, perhaps contributing to the adverse events.

A recently published study in *T. cruzi*-infected mice compared the effect of different BZN doses and the duration of treatment on parasitological cure in both acute and chronic infection. A dose of 30 mg/kg (30% of the standard dose in mice) during 10 and 20-day treatments showed a 67 and 100% cure rate in chronic infections, respectively [26]. In contrast, acute infections showed substantially lower cure rates with either reduced doses or duration of treatment. These results support the possibility of reducing treatment duration and drug dose in chronic infections, as suggested by the authors.

In another prospective population PK study, the authors investigated 39 patients receiving 5 mg/kg daily doses of BZN for 60 days, then built and compared simulated models of 5 mg/kg daily and 2.5 mg/kg daily [27]. Assuming an acceptable BZN plasma concentration range of 3–6 mg/l, the median concentration with the standard dose was above 6 mg/l in all cases. With half doses, all cases showed concentration always within the 3–6 mg/l range. These results support the views of other authors, suggesting that standard treatment protocols using BZN might be significantly overdosing patients and that studies with lower doses in adults might be justified [18, 25].

Thus, in summary, there is currently no experimental evidence in humans evaluating whether BZN treatments that are shorter or lower dose than 5 mg/kg daily for 60 days have a non-inferior trypanocidal effect with fewer adverse events. Observational, animal, and PK studies suggest that the standard scheme may be overdosing patients unnecessarily and increasing adverse events. RCTs testing alternative BZN courses are therefore a priority.

### Ongoing studies

To our knowledge, two RCTs that compare different BZN treatment schemes combining different length and dosage are ongoing. The BENDITA trial compares a BZN course of 300 mg daily for 30 days with the standard course of 300 mg daily for 60 days in *T. cruzi* seropositive subjects with chronic indeterminate phase and positive PCR (S. Sosa-Estani, personal communication). The MULTIBNZ trial compares BZN 400 mg daily for 15 days, BZN 150 mg daily for 60 days, and BZN 300 mg daily for 60 days in 240 adults with and without organ compromise and positive PCR [28].

## Methods/ study design

The format of this section is adapted from the “Methods” section of the CONSORT checklist for non-inferiority randomized controlled trials [29].

## Overview

**Fig. 1:**
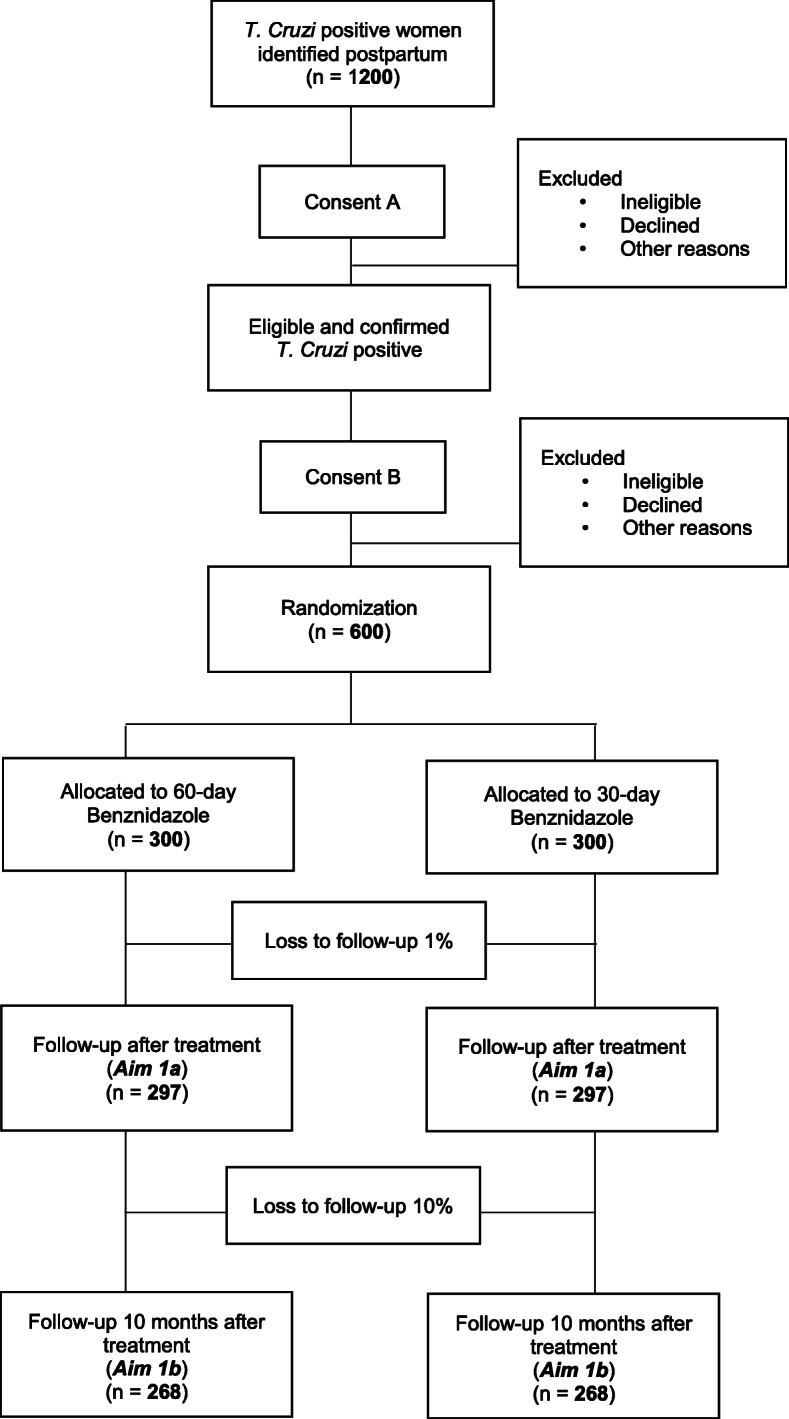
Study design

We are proposing to perform a double-blinded, non-inferiority randomized controlled trial comparing short low dose 30d/150 mg BZN vs. 60d/300 mg BZN. We will recruit not previously treated *T. cruzi* seropositive women with a live birth during the postpartum period in Argentina, randomize them at 6 months postpartum (to avoid interfering with breastfeeding), and follow them up (Fig. 1).

Screening for anti-*T. cruzi* antibodies during antenatal care is standard of care in Argentina [14]. Two serological tests, indirect haemagglutination antibody assay (IHA) and enzyme-linked immunosorbent assay (ELISA) are routinely performed, and mothers are classified as seropositive if they are positive for both. The information is registered on antenatal cards and/or clinical histories. If case information is not available at delivery, serological tests are routinely performed on cord blood. We will identify seropositive mothers with at least one positive or indeterminate test and obtain written informed consent (Consent A) to interview them and to collect maternal blood as soon as possible after delivery. For mothers < 16, we will obtain written assent and written consent from at least one parent or guardian (Assent A and Parental Consent A). If the woman accepts to participate, we will interview her and verify her eligibility. If the mother is eligible, we will collect maternal blood. We will verify the *T. cruzi* seropositivity of mothers with positive antenatal serology by performing an additional IHA and ELISA (Wiener laboratories, Rosario, Argentina). Mothers will be confirmed as seropositive if they are positive for both tests.

Mothers confirmed as seropositive will be visited at home 4–8 weeks postpartum, will be invited to participate in the randomized controlled trial, and will be requested to provide written informed consent (Consent B) specific to trial participation. For mothers < 16, we will obtain written assent and written consent from at least one parent or guardian (Assent B and Parental Consent B). Before treatment, we will perform examinations recommended by the Argentine standards of care, including: EKG, echocardiogram, and chest X-ray [14]. We will also perform complete blood count (CBC), kidney function tests, and liver function tests before treatment and at least once during treatment. Modern contraceptive methods will be provided during treatment. During treatment, we will monitor participants at least once a week for adverse events and adherence and collect DBS for measuring BZN blood levels during the first 30 days of treatment. We will collect blood for *T. cruzi* PCR immediately before treatment, at the end of the treatment (30 and 60 days), and 10 months after treatment.

## Outcome measures

### Primary hypotheses/primary outcomes

To measure the effect of BZN 30d/150 mg compared to 60d/300 mg preconceptional treatment on parasitic load measured by the frequency of positive PCR (primary outcome) and by real-time quantitative PCR (qPCR), immediately (Specific Aim 1a) and 10 months (Specific Aim 1b) after treatment.

Hypothesis 1a: the frequency of positive PCR and the parasitic load measured by qPCR immediately after BZN 30d/150 mg will be non-inferior (Non-Inferiority (NI) margin for PCR: 10% absolute difference) to BZN 60d/300 mg.

Hypothesis 1b: the frequency of positive PCR and the parasitic load measured by qPCR 10 months after BZN 30d/150 mg will be non-inferior (NI margin for PCR: 9% absolute difference) to BZN 60d/300 mg.

### Secondary hypotheses/secondary outcomes

To measure the frequency of serious adverse events and/or any adverse event leading to treatment interruption of BZN 30d/150 mg compared to 60d/300 mg.

Hypothesis 2: the frequency of serious adverse events and/or any adverse event leading to treatment interruption will be 50% lower with BZN 30d/150 mg than with BZN 60d/300 mg.

## Study population and procedures

### Study sites and population

Participating sites: include public health facilities in three endemic provinces in Northern Argentina: Chaco, Santiago del Estero, and Tucumán (Table 1). Women giving birth in these settings have a median age of 23 years old (interquartile range 19–29) [30]. We are planning on enrolling women during a two-year period.

**Table 1:** Participating health facilities: eligible women in 2015

Maternity health facility	Deliveries (N)	*T. cruzi* + (n)	*T. cruzi* + (%)
Hospital J.C. Perrando (Chaco)	5820	186	3.2
Hospital Regional Dr. Ramón Carrillo (Santiago del Estero)	6204	180	2.9
Centro Integral de Salud La Banda (Santiago del Estero)	2720	131	4.8
Instituto de Maternidad y Ginecología Nuestra Señora de las Mercedes (Tucumán)	8692	130	1.5
**Total**	**23,436**	**627**	

### Inclusion and exclusion criteria

#### Inclusion criteria:

- Written informed consent if 16 or older; for mothers < 16, we will require written assent and written consent from at least one parent or guardian.
- *T. cruzi* seropositivity confirmed by at least two positive tests.
- Live birth.

#### Exclusion criteria:

- Women residing outside of the provinces of Chaco, Santiago del Estero, or Tucumán.
- Previous trypanocide treatment (BZN or Nifurtimox).
- Female sterilization; no intention to use modern contraception methods during treatment.
- Positive pregnancy test.
- History of severe alcohol abuse within 2 years; renal insufficiency.

### Sampling, recruitment, and screening procedures

We will identify not previously treated *T. cruzi* seropositive women (with at least one positive or indeterminate test) with a live birth during the postpartum period and obtain written informed consent (Consent A) to interview them and to collect maternal blood as soon as possible after delivery. For mothers < 16, we will obtain written assent and written consent from at least one parent or guardian (Assent A and Parental Consent A). If the woman accepts to participate, we will verify her eligibility. If she is eligible we will collect maternal blood and will verify *T. cruzi* seropositivity by two tests: ELISA and IHA. Mothers confirmed as seropositive will be visited at home 4–8 weeks postpartum, will be invited to participate in the randomized controlled trial, and will be requested to provide written informed consent (Consent B) specific to trial participation. For mothers < 16, we will obtain written assent and written consent from at least one parent or guardian (Assent B and Parental Consent B).

## Other procedures

### Baseline clinical assessment

Between the fourth to sixth months postpartum, we will perform examinations recommended by the Argentine standards of care, including EKG, echocardiogram, and chest X-ray [14]. We will use the Kuschnir classification (0-III) of Chagas disease severity [14, 31]. We will also perform CBC, kidney function tests, liver function tests, and pregnancy tests. These procedures will be done by the Clinical Care Unit (CCU) specifically organized for this study (see Clinical care during treatment).

## Study medication

### Procurement of study drugs, obtaining placebo, development of interventions

We will use a preparation of BZN that is commercially available in Argentina: Abarax® 50 mg tablets from ELEA laboratories (Buenos Aires, Argentina). ELEA laboratories will prepare the placebo tablets, which will be identical to the drug tablets in aspect and taste. We will purchase the drug and placebo at full cost. Samples of 10 tablets of each lot of drug and placebo will be sent to the University of California San Diego (UCSD) for quality control (see Laboratory analysis).

## Randomization procedures

The biostatistician (Dr Shaffer) from the Tulane School of Public Health and Tropical Medicine will generate a random allocation sequence using computer-generated random numbers in balanced blocks of variable sizes, stratified by site. ELEA laboratories (Argentina) will prepare the treatment and placebo tablets. Kits with the two treatment regimens: the 60d standard treatment and the 30d treatment completed with a 30d placebo will be prepared by a packaging company in Argentina. Kits will be identical and will be assigned a lot number, expiration date, and a tracking number (alpha-numeric, with check digit). Treatment allocation will be carried out using an internet-based randomization service (randomize.net) [32]. Each health facility trial unit will have a computer connected to internet provided by the study. Participant eligibility criteria will be entered and confirmed in REDCap and then the participant data will be entered in randomize.net, which will assign a uniquely numbered kit. The system maintains the capacity for managing the stock of kits, assigning replacement kits, or reporting emergency unblinding, if needed.

## Informed consent/assent

All *T. cruzi* seropositive women who are delivering a live newborn at the four participating health facilities will be sought for inclusion as potential study subjects. Women who decline to sign a consent/assent form, pregnant women, women who previously received trypanocidal treatment, women with history of severe alcohol abuse, women with renal insufficiency and women residing outside of the follow-up areas will be excluded.

Participating sites include: Hospital J.C. Perrando (Chaco), Hospital Regional Dr. Ramón Carrillo (Santiago del Estero), Centro Integral de Salud La Banda (Santiago del Estero) and Instituto de Maternidad y Ginecología Nuestra Señora de las Mercedes (Tucumán). The protection of human subjects has been adequately addressed among the collaborating institutions at a level that is at least as high as that documented at the applicant organization, the Tulane University School of Public Health and Tropical Medicine (SPHTM).

Participation in this research is absolutely voluntary. This study will be conducted following strict guidelines for the protection of the rights of human volunteers. We will identify seropositive mothers and obtain written informed consent (Consent A) to interview them and to collect maternal blood as soon as possible after delivery. For mothers < 16, we will obtain written assent and written consent from at least one parent or guardian (Assent A and Parental Consent A). If the woman accepts to participate, we will interview her and verify her eligibility. If the mother is eligible, we will collect maternal blood. We will verify the *T. cruzi* seropositivity of mothers with positive antenatal serology by performing an additional IHA and ELISA (Wiener laboratories, Rosario, Argentina). Mothers will be confirmed as seropositive if they are positive for both tests.

Mothers confirmed as seropositive will be visited at home 4–8 weeks postpartum, will be invited to participate in the randomized controlled trial, and will be requested to provide written informed consent (Consent B) specific to trial participation. For mothers < 16, we will obtain written assent and written consent from at least one parent or guardian (Assent B and Parental Consent B).

Informed consent/assent forms must be signed by all participants. The consent/assent forms will clearly state the study purpose, eligibility criteria and protocol of the study, the potential benefits and risks of participation, and participants’ rights to refuse to participate or withdraw from the study. Additionally, the consent and assent forms and any other materials will be available in both Spanish and English.

If a woman declines to participate, no data will be collected.

## Confidentiality

Data will be collected on paper forms or tablets, and a study identification numbering system (alpha-numeric, with check digit) will be designed specifically for this study. Freezer-safe barcode or QR code labels (Brady, Milwaukee, WI) will be pasted on each of the biological samples for identification. An inclusion form will be used to collect data during participants’ enrollment. Names and other personal identifiers will be recorded in the inclusion form header, which will be redacted from the main body containing data. Each participant will be identified with a study label number. The inclusion form body will be entered into REDCap at each site; names and other personal identifiers will not be entered. This system will ensure that personal identifiers are restricted to the study site and granted only via secure access through the site coordinator.

## Management and retention of study populations

### Clinical care during treatment

At each participating health facility, we will set up a CCU with at least one clinician in charge, including a cardiologist, who will conduct the clinical assessments and will provide their clearance for the women to be randomized. The participating health facilities have the necessary equipment to perform these procedures, and the study will provide all necessary supplies. All tests and diagnostic procedures results will be securely stored at the CCU. The CCUs will also provide clinical care during treatment to the participating women. This study organization has been successfully used in cardiovascular risk factors cohort studies enrolling 8000 adults in Argentina, Chile, and Uruguay and conducted by the Instituto de Efectividad Clínica y Sanitaria (IECS) [33].

Treatment will be prescribed and monitored at each health facility CCU following the Argentine standards [14]. Before the start of the treatment, we will provide participants with their chosen contraceptive methods. During treatment, we will monitor participants at least once a week by phone or SMS, and at biweekly health facility or home visits, for adverse events and adherence. CBC, kidney function tests, and liver function tests will be performed at least once during treatment.

### Follow-up

Women will be followed up until the end of the study follow-up period, 10 months after treatment. To this purpose, a dedicated team in each province will coordinate the follow-up. To maintain updated contact details, participants will be contacted every 2 months by SMS or phone calls. Participants will receive a home visit or a health facility appointment at 10 months after treatment.

### Reimbursements

Enrolled women will not receive any payment for participation in this research. Only travel expenses will be covered.

## Study interventions

### Intervention treatments

We will use a preparation of BZN that is commercially available in Argentina: Abarax® 50 mg tablets from ELEA laboratories (Buenos Aires, Argentina). ELEA laboratories will prepare the placebo tablets, which will be identical to the drug tablets in aspect and taste. We will purchase the drug and placebo at full cost. Samples of 10 tablets of each lot of drug and placebo will be sent to UCSD for quality control (see Laboratory analysis).

Intervention group: BZN short course low dose scheme will be 150 mg per day for 30 days. The treatment will start with the active drug and then placebo; two 50 mg tablets and one placebo tablet in the morning and one 50 mg tablet and two placebo tablets in the evening for the first 30 days. The last 30 days will be three placebo tablets in the morning and in the evening.

Control group: BZN standard course will be 300 mg per day for 60 days, which is similar to the dose used in the second phase of the BENEFIT trial [17]. The drug will be administered orally in two doses per day: three 50 mg tablets in the morning and in the evening for 60 days.

This order will allow both trial arms to start with the active drug and prevent the risk of unblinding that may happen at the occurrence of adverse events in the first 30 days.

### Protocol deviations

A specific report will be produced to account for potential protocol deviations after each phase of the study. The sponsor, the Tulane Institutional Review Boards (IRB), and the local IRBs will be notified of any protocol deviations.

### Adherence monitoring

To monitor adherence, we will use self-report, with a single item visual analogue rating scale performed at the visits to the CCU [34]. Additionally, we will collect DBS for measuring BZN blood levels during the first 30 days of treatment.

## Adverse events

The risks associated with this protocol involve the adverse events (AE) caused by the BZN that were included as secondary outcomes: dermatitis, which usually occurs during the first weeks, peripheral neuropathy, which seems to be related to the cumulative dose and may take months to resolve, and gastrointestinal effects, including vomiting and pain; other severe adverse events, although infrequent, are bone marrow depression, toxic hepatitis, and lymphomas. These adverse events are considered in the secondary outcomes of this study.

Other immediate risks associated with this protocol involve the drawing of venous blood. Occasionally, subjects can feel faint or light-headed, but this is rare and passes by having the subject lie down for a few minutes. Infection is a potential risk, but this risk will be minimized as all specimens will be obtained aseptically by trained personnel.

## Reporting procedures

AEs and serious adverse events (SAEs) will be reported and compiled at each site. They will be forwarded to the Study Data Center (SDC) and to the Principal Investigators (PIs), Pierre Buekens, MD, PhD, in New Orleans, Louisiana, USA, and Maria Luisa Cafferata, MD, in Buenos Aires, Argentina. The sponsor and local IRBs will be notified of any adverse events (when is applicable). Adverse events will be documented in the annual report.

## Measurement methods - variables

### Description of biological clinical and socio-demographic measures and sources

#### Maternal characteristics at enrollment

- A confirmed seropositive mother will have at least two positive tests on maternal blood [35];
- Date of delivery (day/month/year) of a live birth (a baby born with any sign of life, irrespective of gestational age);
- Date of birth (day/month/year); maternal age (years);
- Educational level (completed years);
- Parity (including current birth); reproductive history (year of occurrence): previous pregnancies, abortions, stillbirths, live births, infant’s or children’s death; last menstrual period (LMP) (day/month/year);
- Smoking status (yes/no); severe alcohol abuse (yes/no): 4 or more drinks on any day or 8 or more drinks per week [36] maternal weight (kg); maternal height (cm); Body Mass Index (BMI) (kg/m2);
- Human Immunodeficiency Virus (HIV) serology (according to tests performed routinely in each health facility) (positive/negative);
- Screening for *T. cruzi * (day/month/year); previous trypanocide treatment (BZN or Nifurtimox) (yes/no);
- Household location and municipality; household latitude and longitude (decimal degrees).

#### Women’s characteristics before and during preconceptional treatment

- Contraception method: implant, copper intrauterine device, hormonal intrauterine system, injectable, hormonal contraceptive pill, barrier methods;
- Renal insufficiency (serum creatinine > 2.5 mg/dL or 200 μmol), hepatic insufficiency (AST/ALT >3x normal) [17], EKG, echocardiogram, and chest X-ray (BENEFIT trial criteria [17] and Kuschnir classification (0-III) of Chagas disease severity [14, 31];
- Adherence to treatment: single item visual analogue rating scale (0–100%) [34] and BZN DBS concentration (μg/mL); other medications (name, dosage);
- Adverse events: dermatitis (yes/no), gastrointestinal (yes/no), severe leukopenia (< 2500 white blood cells/μL), peripheral neuropathy (yes/no), others (description).

## Data sources

### Clinical data, socio-demographic data, and geographical data

#### Clinical data and socio-demographic data

Data will be collected from the medical histories and by interviewing the mothers. Study personnel will be trained in abstracting medical histories. Participating health facilities use the format of the Perinatal Information System of Pan American Health Organization (PAHO)/World Health Organization (WHO) [37]. Data on CBC, kidney function tests, liver function tests, EKG, echocardiogram, and chest X-ray will be collected by the CCUs.

#### Geographical data

Street addresses will often be unreliable in the areas where participating women live; thus, detailed information will be collected at enrollment:

- Household location (addresses will be registered if available);
- Municipality of residence. Interviewers will have a list of municipalities available during the interview and verify that the name of the municipality is valid;
- Phone numbers of the participants.

During the first home visit, considerable effort will be made to locate the household, involving key informants, if needed. Once located, we will use GPS to map the location of the household. The latitude and longitude of the main entrance will be determined after obtaining the appropriate GPS signal, and additional characteristics of the household will be noted. To facilitate future household visits, the GPS coordinates will be entered into a GIS database (ArcView Geographic Information Systems [ArcGIS] for Desktop Version 10.3 or higher, Environmental Systems Research Institute, Redlands, CA).

## Schedule of data collection

### Screening

Before delivery, we will identify seropositive mothers with at least one positive or indeterminate test and obtain written informed consent (Consent A) to interview them and to collect maternal blood as soon as possible after delivery. For mothers < 16, we will obtain written assent and written consent from at least one parent or guardian (Assent A and Parental Consent A). If the woman accepts to participate, we will interview her and verify her eligibility. If the mother is eligible, we will collect maternal blood. We will verify the *T. cruzi* seropositivity of mothers with positive antenatal serology by performing an additional IHA and ELISA (Wiener laboratories, Rosario, Argentina). Mothers will be confirmed as seropositive if they are positive for both tests.

If the mother has a live birth, is *T. cruzi* positive confirmed by at least two positive tests, did not receive previous *T. cruzi* treatment, and lives within the study’s follow-up area, then she will be eligible for enrollment in the study.

### Enrollment and follow-up

Mothers confirmed as seropositive will be visited at home 4–8 weeks postpartum, will be invited to participate in the randomized controlled trial, and will be requested to provide written informed consent (Consent B) specific to trial participation. For mothers < 16, we will obtain written assent and written consent from at least one parent or guardian (Assent B and Parental Consent B).

Before treatment, we will perform examinations recommended by the Argentine standards of care, including EKG, echocardiogram, and chest X-ray [14]. We will also perform CBC, kidney function tests, and liver function tests before treatment and at least once during treatment. Pregnancy tests will be performed before and during treatment. Modern contraceptive methods will be provided during treatment. During treatment, we will monitor participants at least once a week for adverse events and compliance with the study medication. We will plan a visit every 2 weeks at the health facility or at home. During the first 30 days of treatment, we will monitor adherence and will collect DBS for measuring BZN blood levels. We will collect blood for *T. cruzi* PCR immediately before treatment, at the end of the treatment (30 and 60 days), and 10 months after treatment (Table 2).

**Table 2:** Summary of procedures

Time	Source	Sample	Tests to perform	Storage requirements
Postpartum period (Consent/ Assent A)	Maternal venous blood	5 ml EDTA for plasma	ELISA and IHA	Filter paper 4 °C Plasma/serum − 20 °C
4–8 weeks postpartum	Home visit to provide consent to be randomized (Consent/Assent B)			
Before randomization	Clinical assessment			
6 months postpartum	Randomization to BZN 30d/150 mg (intervention arm) or BZN 60d/300 mg (control arm)			
Before treatment	Maternal venous blood	5 ml	PCR	10 ml blood-Guanidine 4 °C
Once a week	Monitoring adverse events			
During treatment	Maternal finger prick/ venous blood	DBS	BZN concentration	Filter paper −20 °C
End of treatment	Maternal venous blood	5 ml	PCR	10 ml blood-Guanidine 4 °C
10 months after treatment	Maternal venous blood	5 ml	PCR	10 ml blood-Guanidine 4 °C

### Early termination visit

Subjects may withdraw voluntarily from participation in the study at any time. However, we estimate that the loss to follow-up will be lower than 5%. In the event of early termination, we will attempt to conduct the follow-up visit procedures early, if the participant is willing.

### Questionnaire administration

Data will be collected on paper forms or tablets, and a study identification numbering system (alpha-numeric, with check digit) will be designed specifically for this study. Freezer-safe barcode or QR code labels (Brady, Milwaukee, WI) will be pasted on each of the biological samples for identification. An inclusion form will be used to collect data during participants’ enrollment. Names and other personal identifiers will be recorded in the inclusion form header, which will be redacted from the main body containing data. Each participant will be identified with a study label number. The inclusion form body will be entered into REDCap at each site [38]; names and other personal identifiers will not be entered. This system will ensure that personal identifiers are restricted to the study site and granted only via secure access through the site coordinator.

## Collection of biological samples/shipping

### Blood samples

We will collect blood for *T. cruzi* PCR immediately before randomization as a baseline measurement. For Specific Aim 1, we will collect blood after the end of the treatment with the active drug in each arm (as treatments are masked, we will collect blood in both arms at 30 and 60 days after randomization), and at 10 months after treatment. For Specific Aim 2, we will collect blood for CBC, kidney function tests, and liver function tests before treatment and at least once during treatment. In addition, we will collect information on parasitological and serological examinations routinely performed among infants born alive to a *T. cruzi* seropositive mother according to national policies, including direct parasitological examination on cord blood [14].

We estimate from our previous studies that less than 1% of the samples will be unusable.

Serology: 5 ml of maternal venous blood will be collected in EDTA tubes for plasma.

PCR: 5 ml of maternal venous blood will be collected in tubes with 5 ml guanidine-HCl 6 M, EDTA 0.2 M (pH 8).

BZN measurement: DBS will be collected on Whatman 903 Sample Collection Cards (GE Healthcare Bio-Sciences, Pittsburgh, PA) or equivalent.

### Storage of biological samples

Consent/assent forms will include the authorization to participating study institutions to store blood specimens for up to 10 years for confirmatory studies related to the primary outcomes, and an opt-out option allowing the use of stored specimens for other confirmatory studies (including diagnosis of co-infections) and for potential human genetic studies linked to mother-to-child transmission of *T. cruzi*. This is similar to our previous study, in which less than 1% of the participating women did not accept the storage.

Blood samples will be stored in part on filter paper kept in plastic bags with a desiccant at 4 °C until use, and additional plasma and/or serum samples will be frozen at or below − 20 °C until use. Freezing-defreezing cycles of plasma and/or serum aliquots will be kept to a minimum. The blood samples to be stored for PCR will be collected in tubes with guanidine-HCl 6 M, EDTA 0.2 M (pH 8) and kept at 4 °C until processed.

Aliquots of each blood sample will be stored in-country. In addition, aliquots of frozen plasma and/or serum will be shipped on dry ice to Tulane University and stored at the SPHTM freezer farm, located in a safe area at the Tulane National Primate Research Center, 35 miles north of New Orleans. Aliquots of blood on guanidine will also be stored in-country and at the Tulane SPHTM.

Freezer-safe barcode or QR code labels (Brady, Milwaukee, WI) will be pasted on each of the biological samples and will be scanned to document the location of the samples. Temperatures of freezers, refrigerators, and the storage place for study medication will be monitored by digital temperature data loggers (SM320, Dickson, Addison, IL, or equivalent). The SDC will manage the barcode and temperature data (see Data collection and management).

### Shipping

Each site will ship tubes containing blood/guanidine/EDTA mixtures (10 mL), and labeled tubes containing plasma and/or serum, to the Laboratorio de Biología Molecular de la Enfermedad de Chagas (INGEBI) in Buenos Aires by World Courier or similar door to door service. Such sending and shipping to INGEBI should not raise problems, according to World Courier. INGEBI will ship the tubes to the SPHTM in New Orleans, Louisiana, USA.

Each site will ship DBS filter papers to INGEBI in Buenos Aires, and INGEBI will ship the samples to the USD, California, USA.

For quality assessment, INGEBI will ship tubes containing blood/guanidine/EDTA mixtures (10 mL) to the Instituto Nacional de Parasitología (INP) or a similar reference laboratory in Buenos Aires for qPCR and PCR.

Labeled tubes containing blood/guanidine/EDTA mixtures (10 ml/tube) will be stored at 4 °C until shipped to Buenos Aires and New Orleans, but the shipping can be done at ambient temperature.

Labeled tubes containing plasma and/or serum will be stored at or below − 20 °C until shipped to Buenos Aires and New Orleans, and the shipping will be done on dry ice or similar. DBS filter papers will be stored at or below − 20 °C until shipped to Buenos Aires and San Diego, California.

## Biosecurity safety procedures

All blood sample extraction procedures will comply with biosecurity safety procedures. All laboratory personnel will be trained in handling biohazardous materials. This training will be conducted before the study begins. “Sharps” boxes for appropriate storage of needles and other sharps will be located in the laboratory and appropriately discarded. Biohazardous materials will be discarded in appropriately labeled receptacles. They will be autoclaved and then discarded in appropriate biohazardous waste facilities.

## Laboratory analysis

Investigators and staff performing the laboratory procedures will be blinded for the BZN treatment schemes status and for other characteristics of the participants. Conventional PCR and qPCR will be performed independently using the same methodologies in two different laboratories: INGEBI and the SPHTM. External quality assessment will also be conducted at the SPHTM for serology and at the INP or a similar reference laboratory for conventional PCR and qPCR.

### Serology

Samples will be analyzed at each of the four enrolling health facilities with two commercial tests (Wiener laboratories, Rosario, Argentina): an ELISA prepared with recombinant *T. cruzi* antigens (Chagatest ELISA recombinant, version 3.0 or higher) and an IHA test, according to the manufacturer’s protocols.

### DNA extraction and conventional PCR

We will use the following procedures, or equivalent ones. DNA will be extracted using Qiagen DNA extraction kits (or equivalent). The concentration and quality of DNA will be measured at 260/280 nm. The presence of *T. cruzi* DNA in purified DNA extract will be tested by two conventional PCRs using primers amplifying nuclear microsatellite DNA repeats (SatDNA) (Tcz1/Tcz2, giving amplicons of 182 pb) and kinetoplastic DNA repeats from minicircle domains (kDNA) (Tc121/Tc122, giving amplicons of 320 pb). Each sample will be also analyzed for the human β-globin fragment to verify the integrity of the DNA extracted and the lack of PCR inhibitors. PCR products will be separated on 2% agarose gels and visualized with ethidium bromide or SYBR® Safe DNA Gel Stain. Those samples demonstrating the specific bands for one or both molecular targets will be considered *T. cruzi*-positive. All samples will be analyzed in duplicate. The detection limit of such PCRs was estimated to 0.1 to 1 parasite equivalent/mL [39]. Molecular characterization of *T. cruzi* Discrete Typing Units (DTUs) will be carried out using a barcoding approach based on the mini-exon marker, which has been previously standardized, and the PCR conditions improved by our group. We will first amplify the intergenic region of *T. cruzi* mini-exon using an improved multiplex PCR with the primers reported by Souto et al. [40] which allows for identification of TcI and non-TcI DTUs. Also, we have improved the PCR sensitivity detection using a simple PCR with primers TrypME3 and TccH, designed by our group, that produce a larger fragment of 500 bp. All amplicons will be processed for next-generation sequencing, and sequences will be analyzed using Geneious 11 to identify parasite DTUs present in samples.

### Real-time quantitative PCR (qPCR)

We will use the following procedures, or equivalent ones. A validated duplex qPCR method based on TaqMan probes, targeting *T. cruzi* SatDNA (primers cruzi1/cruzi2 and TaqMan probe cruzi3), will be performed with 5 μL of eluted DNA using FastStart Universal Probe Master Mix (Roche Diagnostics GmbHCorp., Mannheim, Germany) in a final volume of 20 μL [41, 42]. Two slight modifications in PCR reagents will be introduced to published procedures: Uracil-DNA Glycosylase (UDG, Thermo Fisher Scientific, Rockford, IL) incorporation to the reaction mix, as a carry-over contamination control, and the use of human RNAse P gene as the internal amplification control using TaqMan RNase P Control Reagents Kit (Applied Biosystems, Foster City, CA) at a final concentration of 0.5X. The amplification will be performed in a CFX96 Touch Real-Time PCR Detection System (BioRad) device or similar and cycling conditions will be as follows: a first step of 2 min at 50 °C and a second step of 10 min at 95 °C, followed by 40 cycles at 95 °C for 15 s and 58 °C for 1 min. All samples will be analyzed in duplicate. The qPCR results will be converted to an estimated parasitic load (parasite equivalents/mL) using an appropriate standard calibration curve.

## External quality assessment

External quality assessment will be performed at the SPHTM for serology and at the INP (Buenos Aires, Argentina) or a similar reference laboratory for conventional PCR and qPCR.

*Proficiency testing*: proficiency testing is defined as a reference laboratory sending unknown samples for testing to a set of laboratories. Five blinded panels, containing seronegative human blood samples spiked with 1, 10, and 100 p/mL of cultured epimastigotes from CL-Brener (TcVI) *T. cruzi* stock and negative controls will be prepared at the INP, INGEBI, or a similar reference laboratory. Blood samples will be immediately mixed with one volume of guanidine-HCl 6 M, EDTA 0.2 M (pH 8), and, after 48 h at room temperature, stored at 4 °C until DNA extraction and PCR/qPCR analysis. Each quality control panel will be at three-month intervals, covering a period of 1 year at the participating laboratories and the reference laboratory.

*Retesting*: retesting refers to samples that have been analyzed being retested by a reference laboratory, allowing for inter-laboratory comparison. Ten percent of samples from INGEBI will be sent to the reference laboratories (SPHTM for serology and INP or a similar reference laboratory for PCR) to be reanalyzed, using the same standard operating procedures (SOP).

### Measurement of BZN on DBS

BZN will be assayed by electrospray ionization mass spectrometry after separation with HPLC. BZN will be detected through monitoring the m/z = 261.3 (M+) transitions to product (daughter) ions 255.6, with collision energies of 125 V.

### Measurement of BZN in study medication

Measurement of BZN in study medication will be performed at the beginning and/or at the end of the study by electrospray ionization mass spectrometry after separation with HPLC. BZN will be detected through monitoring the m/z = 261.3 (M+) transitions to product (daughter) ions 255.6, with collision energies of 125 V. Acceptance criteria will be adapted from the U.S. Pharmacopeial Convention using the content uniformity (CU) method [43].

## Primary and secondary outcome measures

Primary outcome: parasitic load measured by the frequency of positive PCR (primary outcome) and by real-time quantitative PCR (qPCR), immediately and 10 months after treatment.

Secondary outcome: frequency of serious adverse events and/or any adverse event leading to treatment interruption of BZN 30d/150 mg compared to 60d/300 mg.

## Training

### Training study personnel

Training will be performed for all study personnel and will include: training in laboratory procedures, data collection and data management, and ethical issues.

Training of Interviewers, Biological Sample Collectors, and Laboratory Personnel will be done during the preparatory phase, among other activities. This phase will last approximately 12 months. The specific training activities and training materials will be detailed in the Manual of Operations. All personnel involved in the study will be required to obtain certification ensuring that they have met the training objectives. The SDC at IECS will be responsible for developing this certification. The PI and Project Manager will be responsible for overseeing the certification process.

Detailed SOPs will be created describing all study procedures.

### Training in biosafety procedures for blood extraction

Health facility laboratory personnel include laboratory technicians working at the health facility laboratories. All laboratory personnel will be trained in how to handle biohazardous materials. This training will be conducted before the study begins. “Sharps” boxes for appropriate storage of needles and other sharps will be located in the laboratory and appropriately discarded. Biohazardous materials will be discarded in labeled receptacles. They will be autoclaved and then discarded in biohazardous waste facilities.

### Training in data collection

Field staff includes Field Research Coordinators, Follow-up Coordinators, and Clinicians. Detailed SOPs have been drafted to address all procedures to be carried out by the study staff, including consent/assent, reporting of AEs and SAEs to study physicians, and collection of samples and case report form (CRF) completion at delivery, postpartum, and home visits. For specific aspects of data collection, staff will be trained by senior study staff with respect to assessment of the clinical status of the women, collection of blood samples, and completion of relevant CRFs.

### Training in data management and entry

A detailed SOP will be developed for data entry and data management. The Country Data Manager will be trained in the conduct of accuracy and completion checks of CRFs. He/she will also be trained in methods to query the relevant individual completing the source documents and CRFs with respect to missing data, illegible data, or data that seems erroneous. Finally, the data manager will be trained with respect to handling “out of range” prompts encountered by data entry personnel.

Data entry staff will be trained in methods of data entry of the CRFs into the REDCap system. The data manager and data entry staff will receive relevant human subjects training in confidentiality of data and handling and storage of both the study source documents and study CRFs.

### Training in ethical issues

Before beginning any research activities, all study personnel must participate in training on the proper implementation of study procedures and the ethics of conducting research with human subjects specifically addressing administration of informed consent/assent, confidentiality, and participants’ rights, including the right to withdraw from the study at any time, the right to register complaints about the study, etc. The PI will ensure that all study personnel receive the appropriate training. All personnel involved in the study will be required to obtain certification ensuring that they have met the training objectives. The SDC at IECS will be responsible for developing a certification test. The PI and Project Manager will be responsible for overseeing the certification process.

## Data collection and management

### Overview

Data capture and management will be similar to the plan we have used in previously-funded studies and will be coordinated by IECS (Buenos Aires, Argentina). The randomization sequence and interim data analyses will be independently performed by the Tulane SPHTM biostatistician (Dr Shaffer). Data will be collected on paper forms or tablets, and a study identification numbering system (alpha-numeric, with check digit) will be designed specifically for this study. A freezer-safe barcode or QR code label (Brady, Milwaukee, WI) will be pasted on each of the biological samples. An inclusion form will be used to collect data during participants’ enrollment. Names and other personal identifiers will be recorded in the inclusion form header, which will be redacted from the main body containing data. Each part will be identified with a study label number. The inclusion form body will be entered into REDCap at each site [38]. This system will ensure that personal identifiers are restricted to the study site and granted only via secure access through the site coordinator.

### Data capture

The data forms will be entered in REDCap [38] which is a cloud-based, open source application for capturing and maintaining clinical research data. Paper forms of participant who has signed informed consent will be scanned or photographed and regularly uploaded to a secure cloud server. Names and other personal identifiers will not be included. This system will allow for a digital backup of all study data forms. The SDC will manage and link the information from the temperature data loggers in the study freezers, refrigerators, and the storage place for study medication and will utilize graphs and alerts for detecting values out of the expected range. The SDC will also manage the stock information from the scanned biological samples barcodes.

### Reports

For this trial, periodic reports will be prepared for review by a Data Safety and Monitoring Board (DSMB). The DSMB will be composed of an external peer review group that will monitor the study enrollment and results from the study to ensure the safety and ethical treatment of participants (see DSMB section below for composition and duties). Additionally, weekly on-site visits to the participating health facilities will be carried out by a designated Data Manager, who will complete weekly reports and submit them to SDC staff. These staff will hold weekly monitoring calls with each site Data Manager to discuss site reports and will produce weekly summary reports, which will be submitted to the PI and the coordination unit. The PI and SDC staff will perform at least one site visit per year. During these visits, source data and biological samples verification will be performed in 10% of all participants.

After each data collection period, the SDC will produce a summary report including the number of patients who were included in the study period, missing data rates, and inconsistent data rates. These reports will present overall figures for the entire study and will also be stratified by clinic.

An annual summary report will be produced and sent to the Steering Committee. This report will include a summary of the annual figures regarding data quality, protocol violations, and adverse events.

### Study data center

IECS will serve as the Study Data Center, providing a specific data monitoring system, assuring maintenance of high-quality databases, supervising all data collection procedures, and arranging for the most efficient transfer of study data. In addition, the Tulane SPHTM will provide statistical support and perform interim analyses and reports for review by the DSMB.

The SDC at IECS will be responsible for planning, preparing, and monitoring all data collection procedures and will comply with good clinical practices (GCPs) to ensure high quality data. SDC activities will include: preparing data SOPs, training local Data Center staff for data collection procedures, and monitoring sites’ data collection procedures and quality, and ensuring the confidentiality and safety of the data.

### Adverse events

The SDC will incorporate AE and SAE reporting as an essential component of data collection systems and study monitoring. The SDC will prepare SAE reports for reporting to IRBs and the funding source and will implement and maintain an email notification system to promptly alert investigators of SAEs.

## Statistical analysis

### Data analysis plan and statistical methods

Data will be presented as means and standard deviations and frequencies and percentages, as appropriate. A fixed margin analytical approach will be applied [44], and measures of effect will be quantified using risk differences (RD) with 95% confidence intervals (CI). The null hypothesis of non-inferiority will be rejected if the upper bound of the 95% CI for the RD comparison groups lies below the fixed margin.

### Analysis of primary and secondary hypotheses

For Specific Aim 1a, PCR measurements at completion of treatment will be compared with the active drug in each trial arm (30 and 60 days). More specifically, the blood samples taken at 30 days will be used for the 30d course arm, and the sample taken at 60 days will be used for the 60d course arm. Because it has been shown that PCR negative results may revert to positive after treatment [17], this approach was chosen to ensure that the outcomes are not measured at the same time in both trial arms. A more conservative sensitivity analysis will also be carried out using PCR measurement after 60 days for both groups. For the 30d course arm, subjects whose PCR status changes between the 30d and the 60d measurements will be chronicled and reported. For Specific Aim 1b, the comparisons will use the PCR measurement taken at 10 months from the end of the 60-day treatment period. In the event that the aforementioned sensitivity analysis supports a secular trend bias, correcting for any observed bias will be performed in the 10-month analysis accordingly.

The analysis will be carried out separately based on intention-to-treat (considering subjects lost to follow-up as treatment failures) and per-protocol (excluding subjects lost to follow-up or non-compliant) approaches. Per-protocol analyses are recommended in non-inferiority trials as a secondary approach [45]. Since it is expected that part of the effect of the short treatment arm could be mediated by an increase in compliance, a per-protocol analysis controlling for compliance could contribute to disentangle the mechanism of action. For Specific Aim 2, all analyses will only apply intention-to-treat approaches. Patterns of missing data will be examined separately for each trial arm. It is estimated that approximately 10% of PCR results will be missing at 10 months follow-up. Although unlikely, if more than 20% of PCR results in either arm is missing, sensitivity analysis will be conducted on the effect of the missing data on the inference using multiple imputation procedures [46].

If PCR results at baseline show a clinically significant imbalance between groups, generalized linear models will be used to model RDs for the PCR results (primary outcome), including the treatment as the primary predictor. An analysis of covariance (ANCOVA) approach will be used to model qPCR results (secondary outcomes), including baseline parasite equivalents/mL and treatment as predictors. Treatment effects will be summarized by arithmetic and adjusted mean differences in parasite equivalents/mL between the intervention and control groups, with their corresponding 95% CIs. The qPCR results will be considered on a log scale, as their distribution was found to be right skewed in a previous study. To explore whether the treatment affects qPCR over the entire distribution of parasitic load values or primarily through a reduction of high parasitic load values, the skewness of the parasite equivalents/mL distributions will be tested and compared between the treatment groups. Randomization tests will be used to compare the absolute differences in skewness between the two distributions [47]. If significant differences between the distributions are found, qPCR values will be categorized and analyzed based on these categorical values. All data analyses will be carried out using SAS (Cary, NC) v9.3 or higher.

### Sample size

For Specific Aim 1 and following Food Drug Administration (FDA) and CONSORT guidance, we estimated that the NI margin should preserve 75% of the effect of 60d/300 mg (considered as 100%) over placebo (considered as 0%) found in previous trials [29, 44]. The primary outcome is the frequency of positive *T. cruzi* PCR.

We estimated an effect on PCR conversion to negative with BZN 60d of 80 and 30% with placebo. This is supported by: 1) the observed effects of BZN and placebo in the BENEFIT study in Argentina and Bolivia, which showed conversion rates from a positive to a negative PCR of 73.0% in the BZN 60d group and 28.6% in the placebo group at the end of treatment [17] and 2) the therapeutic response to BZN treatment is better in young and healthy subjects than in older subjects with cardiomyopathy (see 1.2.2 Drug of choice for preconceptional treatment). We will enroll mostly healthy young women (median 23 years old), compared to the older subjects (mean 55 years old) with cardiomyopathy participating in the BENEFIT study. We used the 78.4% positive PCR rate at delivery we found in our previous study in Tucumán, Argentina [48].

Based on these figures, we estimated a frequency of positive PCR of 15% (0.78(1–0.8)) after BZN 60d/300 mg and of 55% (0.78(1–0.3)) after placebo. To preserve 75% of the BZN 60d/300 mg effect over placebo (40% absolute rate difference), the NI margin will be 10.0% absolute rate difference. For the 10-month follow-up aim (Hypothesis 1b), we expect that after almost 1 year, the PCR positive rates will be up to 20% higher, as shown in the BENEFIT study [17]. The NI margin preserving 75% of the effect will be 9% in this case. Table 3 shows that we will need to enroll nearly 540 *T. cruzi* seropositive women. With this sample size, we will have adequate power to detect non-inferiority with a NI margin preserving 75% of the effect of the 60d course over placebo if the PCR rates following treatment are higher than expected, or for preserving 70% if a 3% difference in PCR rates between arms is found. To protect from a potential 10% loss to follow-up at 10 months, we will enroll 600 women.

**Table 3:** Statistical power and sample size assessment for specific aim 1a for *n* = 268 subjects per group (*N* = 536 total subjects)

60d^a^ (%)	30d^a^ (%)	Placebo^a^ (%)	Effect size^a^ (%)	Proportion retained	NI margin^a^ (%)	Power (%)
15.0	15.0	55.0	40.0	0.75	10.0	90
18.0	18.0	55.0	34.0	0.75	9.3	80
15.0	18.0	55.0	40.0	0.70	12.0	80

^a^Cells show PCR positive rate

For Specific Aim 2, we postulated a superiority hypothesis. The rate of serious adverse events leading to treatment interruption observed in the BENEFIT trial was 24%. As mentioned above, our population is healthier and younger; thus, we will conservatively assume a 20% rate. We will have more than 90% power to detect a reduction of serious adverse events and/or any adverse event leading to treatment interruption from 20% in the 60d course group to 10% in the short course group (Table 4) and more than 80% power if the rate is 12%. The two-sided Type I error rate was set at α = 0.025 and 0.05 for the NI and superiority hypotheses respectively, and calculations were carried out with the power sample size calculator for a two-sample non-inferiority study with a sampling ratio of one [49].

**Table 4:** Statistical power assessment for specific aim 2 (superiority hypothesis)

60d (%)	30d (%)	Total (N)	Power (%)
20	10	600	95
20	12	600	81

### Interim analysis and study monitoring

The DSMB will be composed of an external peer review group that will monitor the study enrollment and results from the study to ensure the safety and ethical treatment of participants (see DSMB section below for composition and duties). Membership in the DSMB will consist of persons completely independent of the investigators who have no financial, scientific, or other conflict of interest with the study. The committee will be comprised of an odd number of members to preclude voting ties. Written documentation attesting to absence of conflict of interest will be required. The DSMB will include experts in or representatives of the fields of:

- Clinical trial methodology and conduct.
- Biostatistics/epidemiology.
- Clinical medicine.

The overall responsibilities of the DSMB will be to protect the health and safety of trial participants, to assess the benefits and risks of the trial interventions, and to monitor the scientific integrity of the study conduct. The DSMB for this study will act in an advisory capacity to the PI to monitor patient safety and evaluate the efficacy of the intervention. The initial responsibility of the DSMB will be to review the protocol to ensure that the information being collected is sufficient for the DSMB to monitor study safety and ethical treatment. After this review and at periodic intervals during the course of the study, the DSMB responsibilities will be to:

- Review the research protocol, informed consent/assent documents, and plans for data safety and monitoring.
- Evaluate the progress of the study, including periodic assessments of data quality and timeliness, participant recruitment, accrual and retention, participant risk versus benefit, performance of the sites, adverse events, and other factors that can affect study outcomes.
- Consider factors external to the study when relevant information becomes available, such as scientific or therapeutic developments that may have an impact on the safety of the participants or the ethics of the study.
- Protect the safety of the participants.
- Report on the safety and progress of the study.
- Make written recommendations to the funding agency, the PI, and, if required, to the involved IRBs concerning continuation, termination or other modifications of the study based on the observed beneficial or adverse effects of any treatment(s) under study, or low probability of achieving the study objectives.
- Review proposed modifications to the study prior to their implementation.
- When appropriate, conduct interim analysis of efficacy in accordance with stopping rules that are clearly defined in advance of data analysis and have the approval of the SDC.
- Ensure the confidentiality of the study data and the results of monitoring.
- Assist National Institute of Health (NIH) by commenting on any problems with study conduct, enrollment, and sample size and/or data collection.
- Review any serious issues related to the trial.

### Unblinding

The need for unblinding should be extremely rare, as the adverse events related to BZN may lead to interruption of treatment but reverse spontaneously [19]. If, however, unblinding is needed for any reason, the PI will be informed and Dr. Shaffer will be contacted to reveal the treatment. Moreover, at the analysis phase, if the non-inferiority hypothesis is rejected, mothers will be informed of their allocation to the short course or standard course arm, for discussion with their health providers. Independently of the outcome of the study, at the end of the study all women will be informed of their individual PCR results at 10 months follow-up so that they may provide this information to their health providers. The decision to provide this information is the result of a consensus among investigators and clinicians from the participating health facilities.

## Quality Control (QC) and Quality Assurance (QA)

### Overview

A periodic, systematic, and objective validation of the activities developed in each site will be implemented by the Site Data Manager (SDM) and the SDC.

These activities to be completed include:

- To check that all potential eligible women have been screened. The SDM will compare the screening forms with the delivery logbook.
- To verify that eligibility criteria have been met. The SDM will check the forms with the clinical records.
- To verify that all enrolled women signed Consent Form A at the health facility. For mothers < 16, we will verify that the women and at least one parent/guardian have signed Assent Form A and Parental Consent A. The SDM will monitor a 10–15% sample of the enrolled women to verify that they signed the consent/assent form A and were given a copy.
- To verify that all potential eligible women were tested for confirmatory *T. cruzi* serology. The SDM will verify in the laboratory records the test results of all women.
- To verify that contact data of all eligible women who accepted to participate in this stage at health facility were recorded and kept in a safe location.
- To monitor that all enrolled women are followed up at 4–8 weeks postpartum and invited to participate.
- To verify that all enrolled women who accepted to participate signed Consent Form B during the 4–8 weeks follow-up visit. For mothers < 16, we will verify that the women and at least one parent/guardian have signed Assent Form B and Parental Consent B. The SDM will monitor enrolled women to verify that they signed the consent/assent form B and were given a copy.
- To monitor that all enrolled women who accepted to participate in the trial during 4–8 weeks visit were followed up at 6 months after delivery and were randomized.
- To verify that all women have received the clinical assessment before treatment and were monitored during treatment and at the end of treatment.
- To check the status of the samples at the laboratory. The SDM will select the blood samples and will verify their status at the laboratory (labeling and storage).
- To check adequate labeling and storage of all the data collection forms.
- To check that cool chain requirements have been met.

### Selection of study personnel and job descriptions

#### Site Data Manager

The SDM will have the responsibility of monitoring the data collection activities, producing the data collection reports, and performing quality control and quality assurance of the data collection process. The data manager will monitor the health facility data collection activities. The SDM will monitor the quality in laboratory issues.

#### Study Field Coordinator

The study field coordinator will be responsible for the day-to-day operations of the proposed study at each health facility. She/he will be responsible for the safe storage of all the materials of the study (CRFs, consent/assent forms). She/he will also be responsible for the completion of the corresponding CRFs.

#### Data Collector

The data collector will be responsible for selecting the potential eligible women at each health facility, contacting the women, inviting them to participate, and conducting the informed consent process. She/he will be responsible for completing the corresponding CRFs.

#### Laboratory Technicians

The laboratory technicians will be responsible for performing the maternal venous blood extractions needed for confirmatory serologic tests and PCR assay. She/he will be responsible for collecting DBS for measuring BZN blood levels. She/he will be responsible for completing the laboratory data collection forms and solving any errors that are detected in the CRFs.

#### Follow-up Coordinator

The follow-up coordinator will be in charge of implementing the follow-up system for women and collecting pertinent data. She/he will be responsible for inviting women to participate in the trial and asking the women to be randomized at 6 months postpartum. She/he will be responsible for follow-up activities and performing maternal venous blood extraction before treatment, at the end of treatment and at 10 months after treatment. She/he will provide contraceptive methods to the mothers during treatment. During treatment, she/he will monitor participants at least once a week for side effects and adherence and refer the woman to the Clinical Unit if necessary.

#### Clinical Unit Director

The clinical unit director in each site will be responsible for performing the examinations before treatment (EKG, echocardiogram, chest X-ray, CBC, kidney function tests, liver function tests, and pregnancy test) and at least once during treatment (CBC, kidney function tests, liver function tests, and pregnancy test).

### Training procedures

Field personnel will be trained in all the study procedures (see Training study personnel).

### On-site monitoring

Monitoring will be carried out through weekly on-site visits to the participating health facilities by a designated Data Manager, who will complete weekly reports and submit them to SDC staff. These staff will hold weekly monitoring calls with each SDM to discuss site reports and will produce weekly summary reports, which will be submitted to the PI and the coordination unit. The SDC staff will perform at least two site visits per year. During these visits, source data and biological samples verification will be performed in at least 10% of all participants.

### Site visits

A site visit by the SDC staff or the PI of the study will be done at least twice a year. During this site visit the monitoring and data quality processes will be audited:

- Process forms will be checked.
- Informed consent process will be monitored.
- At least a sample of 10–15% of the CRFs will be verified on the Clinical and Laboratory records during the site visit.
- Cool chain requirements of the blood samples will be checked.
- A sample of blood samples will be checked (amount of blood, storage, and labeling).
- Adequate labeling of all data collection forms and blood samples will be checked.
- Safe storage of the data collection forms will be checked.

### Summary reports

All the QC/QA activities will be reported to the SDC. The SDC will summarize these data and will submit them to the PI at Tulane.

## Quality control in laboratory testing

### External quality assessment

External quality assessment will be performed at the SPHTM for serology and at the INP (Buenos Aires, Argentina) or a similar reference laboratory for PCR and qPCR.

### Proficiency testing

Proficiency testing is defined as a reference laboratory sending unknown samples for testing to a set of laboratories. Five blinded panels, containing seronegative human blood samples spiked with 1, 10, and 100 p/mL of cultured epimastigotes from CL-Brener (TcVI) *T. cruzi* stock and negative controls will be prepared at the INP, INGEBI, or a similar reference laboratory. Blood samples will be immediately mixed with one volume of guanidine-HCl 6 M, EDTA 0.2 M (pH 8), and, after 48 h at room temperature, stored at 4 °C until DNA extraction and PCR/qPCR analysis. Each quality control panel will be at three-month intervals, covering a period of 1 year at the participating laboratories and the reference laboratory.

*Retesting*: retesting refers to samples that have been analyzed being retested by a reference laboratory, allowing for inter-laboratory comparison. Ten percent of samples from INGEBI will be sent to the reference laboratories (SPHTM for serology and INP or a similar reference laboratory for PCR) to be reanalyzed, using the same SOPs.

## Study organization

Our steering committee includes the PIs, one co-investigator from the SDC, one co-investigator from Drugs for Neglected Diseases initiative (DND*i*), and one co-investigator from each of the core laboratories. IECS will act as the SDC. The proposed study will require 60 months (5 years) to complete, including 24 months for patient enrollment (Table 5).

**Table 5:**
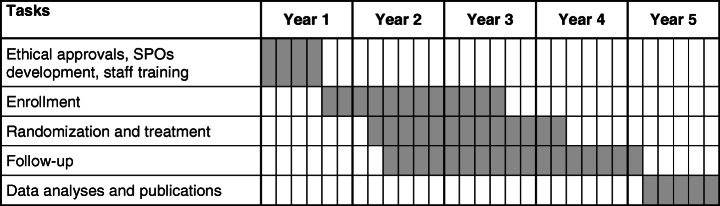
Timeline and tasks

## Human subjects

The described protocol with informed consent/assent will be carried out after approval by the IRB of the Tulane University Health Sciences Center and the IRBs/ethics committees of the respective collaborating institutions, the University of California at San Diego, and IECS and Health Policy, Argentina.

### Description of participants

We will recruit subjects during 24 months in four health facilities in Argentina with a total of 23,436 deliveries in 2015. In each participating health facility, we will recruit not previously treated *T. cruzi* seropositive women with a live birth during the postpartum period in Argentina, randomize them at 6 months postpartum, and follow them up. We plan to invite 600 *T. cruzi* seropositive women of childbearing age to participate, which will include children in the age range of 13–17.

Participating sites include Hospital J. C. Perrando in Chaco province, Hospital Regional Dr. Ramón Carrillo and Centro Integral de Salud La Banda, both in Santiago del Estero province, and the Instituto de Maternidad y Ginecología Nuestra Señora de las Mercedes in Tucumán province. The protection of human subjects has been adequately addressed among the collaborating institutions at a level that is at least as high as that documented at the applicant organization, the Tulane University SPHTM.

Inclusion criteria:

- Written informed consent if 16 or older; for mothers < 16, we will require written assent and written consent from at least one parent or guardian.
- *T. cruzi* seropositivity confirmed by at least two positive tests.
- Live birth.

Exclusion criteria:

- Women residing outside of the provinces of Chaco, Santiago del Estero, or Tucumán.
- Previous trypanocide treatment (BZN or nifurtimox).
- Female sterilization; no intention to use modern contraception methods during treatment.
- Positive pregnancy test.
- History of severe alcohol abuse within 2 years; renal insufficiency.

### Recruitment

Preventive treatment should be theoretically offered either at preconceptional visits or at routine health visits. However, from a public health perspective, this implementation plan collides against two barriers: 1) preconceptional visits are not usual practice in Argentina or other *T. cruzi* endemic countries in Latin America, and around half of the pregnancies are unplanned [50]; and 2) screening for *T. cruzi* infection is not currently standard practice in women of reproductive age who attend routine health visits.

There are no publications reporting the number of women of reproductive age who have received treatment in Argentina. We have requested this information from the provincial Chagas centers in the three provinces where our proposed trial is planned; they have confirmed that no systematic records exist and reported that asymptomatic infected women are very infrequently treated in the public health sector at the current time.

Treating women during the postpartum period is the most feasible approach for preconceptional treatment in Argentina and other endemic countries, because *T. cruzi* serological screening is routinely performed during pregnancy. Deciding to be treated or not is the single most important question facing mothers who have found out they are *T. cruzi* seropositive during a routine antenatal screening.

BZN treatment during breastfeeding results in limited drug transfer into breast milk and seems to have no detectable side effects for the infant [45, 51] but the maternal side effects could cause the interruption of breastfeeding. This reinforces the importance of testing a short treatment scheme that might reduce side effects. Additionally, initiating the treatment at or after 6 months postpartum would be prudent.

### Informed consent/assent

Participation in this research is absolutely voluntary. This study will be conducted following strict guidelines for the protection of the rights of human volunteers. The subjects of the study are *T. cruzi* seropositive women delivering a live birth at the participating health facilities. Written informed consent/assent will be obtained immediately after delivery (Consent/Assent A) and 4–8 weeks postpartum (Consent/Assent B). Informed consent/assent forms must be signed by all participants. The consent/assent forms will clearly state the study purpose, eligibility criteria, and protocol of the study, the potential benefits and risks of participation, and participants’ rights to refuse to participate or withdraw from the study.

Consent/assent forms will include the authorization to participating study institutions to store blood specimens for up to 10 years for confirmatory studies related to the primary outcomes, and an opt-out option allowing the use of stored specimens for other confirmatory studies (including diagnosis of co-infections) and for potential human genetic studies linked to mother-to-child transmission of *T. cruzi*.

Consent/assent forms will also include authorization to be potentially contacted after the study is over for invitations to participate in potential future studies related to *T. cruzi* infection.

## Incentives and other benefits

No incentives will be offered to enrolled women. Transportation costs for the participating women will be provided or reimbursed.

## Publications and presentations

The last 10 months of the project will be dedicated to: 1) conducting the data analysis following a previously prepared data analysis plan; 2) reporting results to participating countries, the funding agency, and other stakeholders; 3) writing and submitting main manuscripts for publication; and 4) disseminating the findings at selected scientific or health management meetings.

## Discussion

Congenital infection is a major source of *T. cruzi* infection, and congenital Chagas disease is associated with severe complications later in life [3]. In consequence of this, it is important that *T. cruzi* seropositive women in reproductive age receive and complete the properly treatment [14]. However, in most cases adherence to treatment is difficult to achieve and it is one of the most common causes that affects its effectiveness.

Particularly in Argentina, the fear of treatment-related side effects limits the implementation of the Argentine guideline recommending BZN standard regimen treatment of *T. cruzi* seropositive women during the postpartum period to prevent transmission in a future pregnancy [14].

We are proposing to evaluate whether a BZN scheme with an overall dose of 4.5 g (150 mg daily for 30 days) is non-inferior to a standard scheme with an overall dose of 18 g (300 mg daily for 60 days), a four-fold reduction. This scheme may work in two ways. First, it has the potential of reducing side effects and treatment interruptions, thus increasing the effectiveness of the treatment through improving patient compliance. Second, this lower dose and shorter scheme may have a comparable trypanocidal effect than the standard scheme. If non-inferior to the standard scheme, and with fewer side effects, this short-low dose scheme for preconceptional treatment of *T. cruzi* infection would be safer to women, shorter, and less costly. The results would impact all women infected by *T. cruzi*, including the large population living in the American countries especially those women from vulnerable populations who are more affected by the disease.

On the other hand, if this scheme proves to be not inferior to the standard treatment, it will have important implications for research purposes, installing a lower limit for the future research of alternative schemes. Also, this new scheme of treatment could be considered by the health authorities and be included into the Chagas disease guideline as a direct public health impact.

Additionally, our focus on the postpartum period is unique, and, if successful, would provide a feasible solution to mothers and their health providers. The trial we are proposing to conduct would be the first controlled trial comparing alternative BZN schemes among women of reproductive age. The other current ongoing trials comparing BZN schemes either do not compare the same schemes or are not specifically conducted among women of reproductive age.

## Data Availability

Not applicable.

## Abbreviations

AEs: Adverse events
BZN: Benznidazole
CBC: Complete blood count
CCU: Clinical Care Unit
CRF: Case report form
DNA: Deoxyribonucleic acid
DSMB: Data Safety and Monitoring Board
ELISA: Enzyme-linked immunoSorbent assay
IECS: Instituto de Efectividad Clínica y Sanitaria
IHA: Indirect haemagglutination antibody assay
INGEBI: Instituto de Investigaciones en Ingeniería Genética y Biología Molecular
INP: Instituto Nacional de Parasitología
IRB: Institutional Review Board
NI: Non-Inferiority
PCR: Polymerase Chain Reaction
PIs: Principal Investigators
PK: Pharmacokinetics
qPCR: Quantitative real-time Polymerase Chain Reaction
RCTs: Randomized controlled trials
SAEs: Serious adverse events
SatDNA: Microsatellite DNA repeats
SDM: Site data manager
SPHTM: Tulane School of Public Health and Tropical Medicine
SOP: Standard operating procedures
UCSD: University of California San Diego
